# Supplementary material for: The interactome of intact mitochondria by cross-linking mass spectrometry provides evidence for coexisting respiratory supercomplexes
Source: Mol Cell Proteomics. 2017 Dec 8;17(2):216–32. doi: 10.1074/mcp.RA117.000470 (PMC5795388; doi:10.1074/mcp.RA117.000470)
Supplement: Supplemental Data [file supp_RA117.000470_133922_0_supp_23978_fzffwf.zip › spectra_annotation/mito_DR_spectra_annotation/499-1-15-1-12-1.pdf]

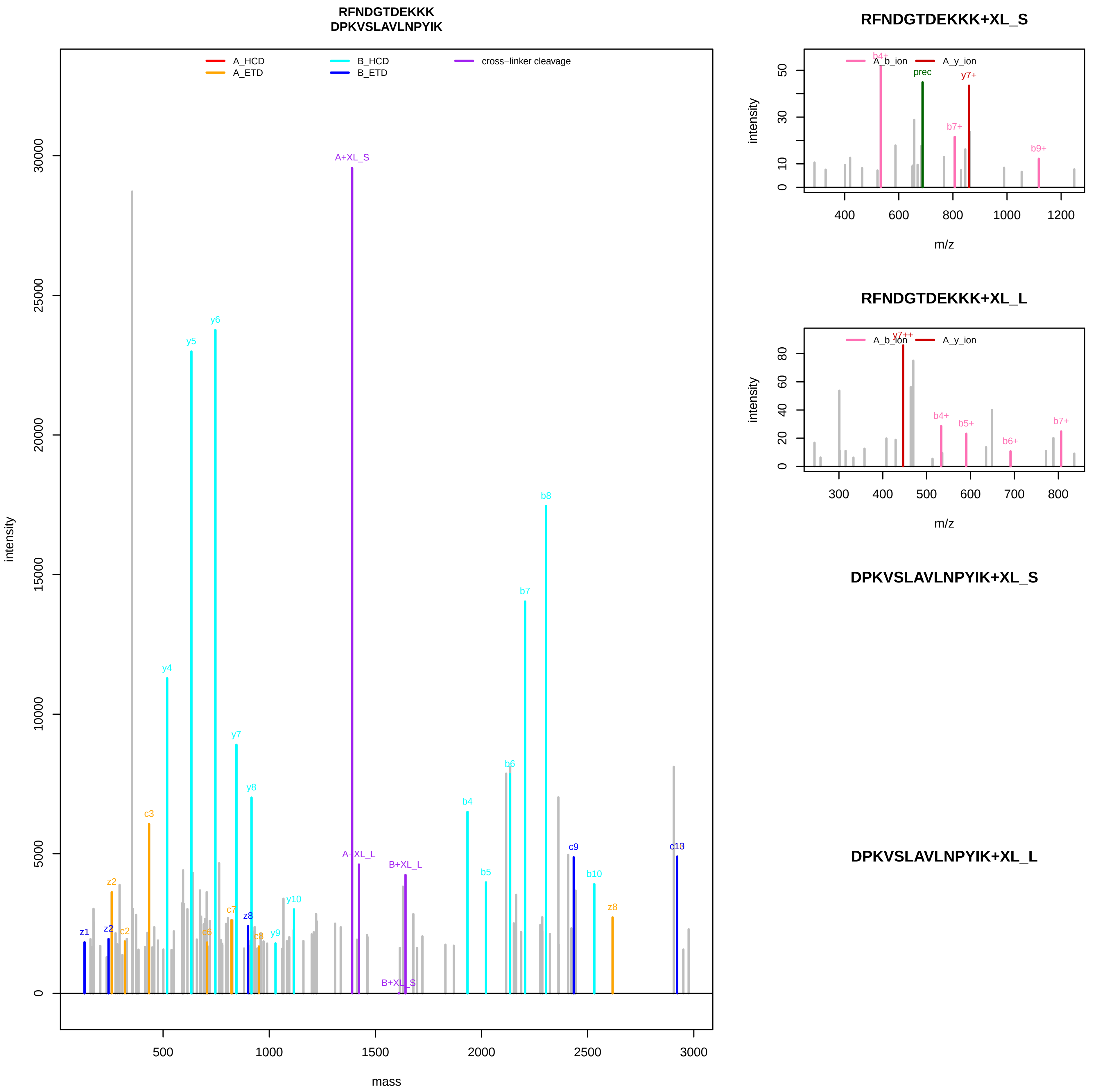
RFNDGTDEKKK
DPKVSLAVLNPYIK
A_HCD
A_ETD
B_HCD
B_ETD
cross−linker cleavage
intensity
mass
0
5000
10000
15000
20000
25000
30000
500
1000
1500
2000
2500
3000
A+XL_S
A+XL_L
B+XL_L
B+XL_S
y4
y5
y6
y7
y8
y9
y10
b4
b5
b6
b7
b8
b10
z1
z2
z2
c2
c3
c6
c7
z8
c8
z8
c9
c13
RFNDGTDEKKK+XL_S
A_b_ion
A_y_ion
b4+
prec
y7+
b7+
b9+
intensity
m/z
0
10
30
50
400
600
800
1000
1200
RFNDGTDEKKK+XL_L
A_b_ion
A_y_ion
y7++
b4+
b5+
b6+
b7+
intensity
m/z
0
20
40
60
80
300
400
500
600
700
800
DPKVSLAVLNPYIK+XL_S
DPKVSLAVLNPYIK+XL_L
